# Variable Influences of Water Availability and Rhizobacteria on the Growth of *Schizachyrium scoparium* (Little Bluestem) at Different Ages

**DOI:** 10.3389/fmicb.2019.00860

**Published:** 2019-05-15

**Authors:** Rhiannon Vargas, Amanda M. Kenney, Teresa Bilinski

**Affiliations:** ^1^Department of Biological Sciences, St. Edward’s University, Austin, TX, United States; ^2^Division of Biology and Biomedical Sciences, Washington University in St. Louis, St. Louis, MO, United States

**Keywords:** PGPR, native grassland species, environmental stress, water limitation, rhizosphere manipulation, rhizosphere microbes

## Abstract

There is significant interest in understanding the role of plant growth-promoting rhizobacteria (PGPR) in alleviating different types of plant stress. *Schizachyrium scoparium* (little bluestem) is a moderately drought tolerant, perennial bunchgrass native to North America. The goal of this experiment was to evaluate whether the addition of a bacterial root isolate in the *Pseudomonas* genus promoted the growth of *S. scoparium* with changes in water availability. *Pseudomonas* are common rhizobacteria and have been shown to improve plant growth. It was hypothesized that plants inoculated with the PGPR strain would have greater growth and health, and would be less affected by shifts in water availability. *Pseudomonas* strains were isolated from the roots of native *S. scoparium* plants. After germination, *S. scoparium* seedlings were subjected to four treatment groups: low water; high water; low water with PGPR; and high water with PGPR. The experiment was run three times with plants at different starting ages; 14-, 28-, and 70-day-old plants. The effects of the water and PGPR treatments were variable between the experimental trials. There were no significant effects of the water treatments on plant growth in Trial 1 (14-day-old plants) or Trial 2 (28-day-old plants), however, there was a significant negative effect of the high watering treatment on the shoot length and biomass in Trial 3. High water availability was significantly associated with greater plant health in Trial 1, but appeared to reduce plant health in Trials 2 and 3. The PGPR treatment appeared to promote root growth and biomass in Trial 2, and was associated with greater plant health in all three trials, especially when paired with the low water treatment. Results from a permutational MANOVA indicate that plant growth was significantly different between the trials due to differences in the starting age of the plants and the duration of the experiments. Thus, methodological choices, such as plant life history stage and experiment duration, may affect the response of plants to PGPR in the rhizosphere. This research provides an insight into the interactions between PGPR and water availability on the growth and health of native plants.

## Introduction

Interactions with both the abiotic and biotic components of a plant’s environment significantly impact its growth and reproduction (Lambers et al., 2008; Suzuki et al., 2014). These include abiotic resources, such as water, temperature, light, and nutrients, as well as other organisms, including competitors, herbivores, pathogens, and beneficial microorganisms. Root-associated microorganisms, also known as rhizosphere microorganisms, form complex, and often beneficial, interactions with plants. They can promote plant growth by shifting biotic conditions in soil, primarily through decreasing infection by microbial pathogens (Cook et al., 1995; El-Sayed et al., 2014). Rhizobacteria may also alter abiotic conditions in the soil environment, for example, through increasing nutrient availability (Richardson et al., 2009; Pii et al., 2015). Likewise, rhizobacterial growth and activity is strongly affected by soil environmental conditions (Schimel et al., 2007; Berg and Smalla, 2009; Philippot et al., 2013; terHorst et al., 2014; Xiao et al., 2017), and plants appear to have a strong selective pressure on the bacteria that colonize the rhizosphere through the production of root exudates (Berg and Smalla, 2009; Ling et al., 2011; Patel et al., 2015; Guyonnet et al., 2018). The complex interactions between plants, rhizobacteria, and the abiotic environment are critical to understanding the many factors affecting plant growth.

Plants have a suite of physiological and morphological mechanisms to cope with abiotic (e.g., drought, flood, high salt) and biotic (e.g., herbivory, attack from pathogens) stress (Lambers et al., 2008). For example, either increasing or decreasing water-use efficiency (the ratio of photosynthesis to stomatal conductance) can improve plant fitness under drought, depending on the timing of drought onset (Heschel et al., 2002; Heschel and Riginos, 2005). Production of secondary metabolites and many proteins can function as defense against herbivores and pathogens (Walling, 2000; Wittstock and Gershenzon, 2002). Morphological examples include structural defense against herbivory, such as spines and pubscence (Hanley et al., 2007), and specialized tissues to promote gas exchange between the soil, root, and shoot in flooded soil, an important trait for flood tolerance (Mommer et al., 2006; Colmer and Voesenek, 2009). Recent research has shown that interactions with beneficial rhizobacteria can also confer resistance to various abiotic stressors such as drought (Kang et al., 2014; Naylor and Coleman-Derr, 2018).

Plant responses to drought influence the function of the root microbiome (Antoun, 2013; Badri et al., 2013). Microbes utilize amino acids, plant hormones, and organic compounds directly released from the plant roots (Henry et al., 2007; Antoun, 2013; Canarini et al., 2016; Calvo et al., 2017). Often the root exudates secreted by plants shift when a plant is under stress (Patel et al., 2015; Naylor and Coleman-Derr, 2018). Stress, for example, caused by high levels of solar radiation, nutrient deficiency, or drought, stimulates the production of 1-aminocyclopropane-1-carboxylate (ACC), a precursor for the plant stress hormone ethylene (Lynch and Brown, 1997; Yang et al., 2009). Some strains of rhizobacteria are able to degrade ACC using the enzyme ACC deaminase, which minimizes the plant’s stress response (Glick, 2004; Zahir et al., 2008; Timmusk et al., 2011; Saikia et al., 2018). Many different plant-derived nutrients promote rhizobacteria growth and activity, which drives a feedback in which bacteria solubilize essential plant nutrients, such as phosphorus and potassium, which in turn drives overall plant productivity (Micallef et al., 2009; Naylor and Coleman-Derr, 2018).

Plant growth-promoting rhizobacteria (PGPR) are usually free-living rhizosphere bacteria that comprise a stable part of the rhizosphere microbial community (Mayak et al., 2004; Grönemeyer et al., 2012; Antoun, 2013; Timmusk et al., 2014). PGPR form close associations with plants which lead to increases in overall plant growth through the production of growth-stimulating phytohormones and metabolites that promote plant growth (Mayak et al., 2004; Belimov et al., 2009; Dimkpa et al., 2009; Timmusk et al., 2014). In addition, PGPR outcompete and suppress pathogens (Cook et al., 1995; Pierson and Pierson, 1996; Whipps, 2001). PGPR also help plants via the synthesis of signaling molecules that trigger protective responses within the plant (Cho et al., 2008; Marasco et al., 2012), which affects plants’ susceptibility to stress. In addition, while undergoing stress plants may excrete higher levels of organic acids, which increases the recruitment of PGPR (Patel et al., 2015). The root architecture of plants, under normal and stress conditions, may also play an important role in recruiting PGPR to colonize root surfaces (Saleem et al., 2018). The dynamics of how rhizobacteria affect plants’ response to stress is highly dependent on environmental factors.

Soil conditions exert a strong influence on the way in which rhizobacteria shape plants’ response to certain stressors. In soils with high levels of contaminants, PGPR decrease the negative effects of toxins on plant growth by degrading the contaminants, and by affecting the expression of plant genes encoding for stress responses (Gurska et al., 2015; Zhang et al., 2017). Soil organic matter concentrations also affect the degree to which PGPR stimulate plant growth. Results from recent studies indicate that soil organic matter and PGPR inoculation have a synergistic effect on plant growth, especially for experiments involving organic matter additions and amendments in degraded soils (Çakmakçi et al., 2006; Mengual et al., 2014). Plants and rhizobacteria, as well as their interactions, are strongly influenced by soil water availability. For example, Lau and Lennon (2011) observed that plant acclimation to drought stress was promoted by shifts in rhizosphere microbial community structure. PGPR promote plant acclimation to water limitation by degrading ACC, thereby dampening plants’ stress response pathways (Saleem et al., 2007; Zahir et al., 2008; Belimov et al., 2009; Saikia et al., 2018). In addition, Sandhya et al. (2009) isolated multiple *Pseudomonas* strains that produced exopolysaccharides which promoted the growth of seedlings in response to drought stress (Sandhya et al., 2009). For older plants, inoculation with PGPR may indirectly alleviate drought stress by stimulating root growth and increasing root surface area, thereby increasing water uptake by plants (Marasco et al., 2013; Wang et al., 2014).

Bacteria in the *Pseudomonas* genus are one of the most dominant and well characterized PGPR in literature, and their role in improving stress tolerance amongst a variety of plant host species has been well-documented (Podile and Kishore, 2006; de Bruijin et al., 2007; Sandhya et al., 2010; Beneduzi et al., 2012; Gera Hol et al., 2013; Vurukonda et al., 2016; and others). *Pseudomonas* is one of the most dominant PGPR genera found natively in soils (Cook et al., 1995; El-Sayed et al., 2014; Wang et al., 2014). *Pseudomonas* are able to colonize the roots of several different plant species, and their root associations with plants enhance root length, shoot length, and biomass under a range of environmental conditions (Timmusk et al., 2014; Oteino et al., 2015; Lally et al., 2017). *Pseudomonas* strains assist plants undergoing stress by producing antibiotic compounds and inducing plant immune defenses, which defend the plant from pathogen attachment and invasion (Cook et al., 1995; Pierson and Pierson, 1996; Chen et al., 2000). Experimental evidence has documented the effectiveness of PGPR in the *Pseudomonas* genus in promoting plant growth in response to water stress (Çakmakçi et al., 2006; Zahir et al., 2008; Sandhya et al., 2009; Gurska et al., 2015; Saikia et al., 2018). *Pseudomonas* strains, such as *P. putida*, likely modulate plant physiology and plant tissue stoichiometry during drought through the production of signaling molecules that shift hormone and antioxidant production (Kang et al., 2014). Thus, research investigating the interactions between PGPR and plants has focused on different aspects of *Pseudomonas* effects on plant growth under a range of conditions. However, a majority of this research has evaluated PGPR effects on crop plants, and there is relatively little known about PGPR effects on wild species, especially grasses.

The goal of this research was to evaluate the individual and synergistic effects of a native *Pseudomonas* rhizobacterium and water availability on the growth of the perennial bunchgrass *Schizachyrium scoparium* (Michx.) Nash (little bluestem). *S. scoparium* is native to grasslands throughout North America and occurs in many different soils and ecosystems across a wide precipitation gradient (Steinberg, 2002; Tober and Jensen, 2013; PRISM Climate Group, Oregon State University, 2015; USDA-NRCS, 2019). Furthermore, *S. scoparium* experiences periodic water limitation in its native range and is moderately tolerant to drought conditions (Mueller and Weaver, 1942; Maricle and Adler, 2011; Tober and Jensen, 2013; Maricle et al., 2015). However, it has historically succumbed to prolonged extreme drought (Weaver et al., 1935; Weaver and Albertson, 1939; Weaver and Albertson, 1943). *S. scoparium* accesses most of its water in relatively shallow soil layers (∼5–50 cm, Eggemeyer et al., 2009; Mueller et al., 2013), and its response to water limitation is to resist the negative effects of drought through physiological adjustments (Hake et al., 1984; Knapp, 1984; Maricle and Adler, 2011; Maricle et al., 2015). Due to its adaptability to various soil conditions, *S. scoparium* serves as an important forage species for livestock and wildlife, and can be used in erosion control (USDA-NRCS, 2002). In addition, the response of *S. scoparium* and other native grass species to a range of environmental conditions, especially with respect to water availability, is especially critical in the face of global change. Climate projections for Central Texas, where this study took place, forecast an increase in extreme drought frequency over the coming decades (Chen et al., 2012; Deng et al., 2018). Thus, it is beneficial to understand the extent of *S. scoparium*’s tolerance to shifts in water availability, as well as the potential for native PGPR strains to affect the response of *S. scoparium* to changing environmental conditions.

This research was designed to test the hypothesis that the addition of a single PGPR *Pseudomonas* strain will cause a significant positive effect on the growth of *S. scoparium* across a range of water availability, and in plants of different ages. Specifically, we predicted that the addition of a PGPR strain at regular intervals will result in improved observable plant health, longer root and shoot lengths, and greater plant biomass compared to plants that did not receive the bacterial addition. In addition, we predicted that the effect of PGPR addition would vary with water availability. Importantly, this study also evaluated the repeatability of these effects across three different trials of a full-factorial greenhouse experiment, while varying the starting age of plants among trials. Results from this research show that supplementing the rhizosphere microbiome with a native PGPR can increase plant growth. However, we observed a high degree of variability in the response of *S. scoparium* to PGPR addition between the trials. Our results illustrate the importance of methodological choices, such as the starting age of plants and experiment duration, in the responses of plants to PGPR addition. Furthermore, most research on plant-microbe interactions has been performed on semi to highly domesticated agricultural plants (Cook et al., 1995; Chen et al., 2000; Belimov et al., 2009; Marasco et al., 2013), which are often ecologically distinct compared to many wild species (Milla et al., 2015). Therefore, studying plant–microbe interactions in a wild species represents an important contribution to understanding how rhizobacteria affect plant growth and mediate response to stress in natural ecosystems.

## Materials and Methods

### Overview of Experimental Design

In order to test the effect of a native *Pseudomonas* rhizobacteria on *S. scoparium* growth under both non-stress and stress conditions, we used a two-way full factorial experimental design with water availability and the addition of a *Pseudomonas* culture as factors. The four treatment groups were: (1) well-watered plants with the addition of uncultured, sterile *Pseudomonas* broth (W); (2) plants watered at a reduced, or drought, level with the addition of uncultured, sterile *Pseudomonas* broth (D); (3) well-watered plants with the addition of a *Pseudomonas* culture grown in *Pseudomonas* broth (BW); and (4) watering at a reduced level and the addition of a *Pseudomonas* culture grown in *Pseudomonas* broth (BD). Three trials were conducted, with sixty plants for each trial and 15 individuals per treatment.

### PGPR Isolation, Characterization, and Growth

Wild samples of *S. scoparium* were collected from Blunn Creek Nature Preserve in Austin, TX, United States (30°13′57.59″ N; Longitude: 97°44′52.20″ W) in order to isolate native PGPR *Pseudomonas* strains from its roots. For the first experimental trial *S. scoparium* samples were collected on May 31, 2016. To isolate the strain used in the second and third experimental trials *S. scoparium* samples were collected on February 26, 2017.

To isolate *Pseudomonas* strains, surrounding bulk soil was removed from the roots. Then the roots were placed in 500 mL of sterilized *Pseudomonas* broth containing 20 g/L enzymatic digest of soybean or tryptone, 1.4 g/L magnesium chloride, 10 g/L potassium sulfate, and 0.025 g/L Irgasan, for 1 h. The roots were then removed from the broth, and the inoculated broth was incubated shaking at 30°C for 24 h. After 24 h, an aliquot of the bacterial culture was transferred to a *Pseudomonas* agar plate using the streak plate method, and was incubated at 30°C for 24 h. A single *Pseudomonas* isolate from this agar plate was transferred to a fresh *Pseudomonas* agar plate in order to obtain a pure culture. The isolates were visualized using the gram staining method and 100× bright field microscopy. In addition, the identity of the isolates as *Pseudomonas* was confirmed using microscopy and the Gen III BIOLOG system (Hayward, CA, United States^1^). The isolates were then used for plant inoculation during the experiment (see section “Experimental Treatments: Water Availability and PGPR”). *Pseudomonas* strain A was isolated using these methods in May 2016. In January 2017 it was not possible to revive this isolate from a glycerol stock stored at -20°C. As a result, *Pseudomonas* strain B was isolated using a different *S. scoparium* collected from the same area of Blunn Creek Nature Preserve in February 2017. Strain A was used for Trial 1, in which the experiment started with 14-day-old plants (Table 1). Strain B was used in Trials 2 and 3, which started with 28- and 70-day-old plants, respectively (Table 1). Following pure culture isolation, strains were preserved as a glycerol stock by adding 500 μl of bacterial culture with 500 μl of 50% sterilized glycerol, and stored at 20°C.

**Table 1:** A summary of the methodological differences between the trials.

Trial	Starting age	Experiment duration	Bacterial strain	Amount of liquid added per addition	Total amount of liquid received during the experiment	Equivalent inches of rain/year
1	14 days	8 days	A	HW: 120 LW: 20	HW: 480 mL LW: 80 mL	HW: 49 in LW: 8 in
2	28 days	25 days	B	HW: 25 LW: 20	HW: 300 mL LW: 240 mL	HW: 10 in LW: 8 in
3	70 days	25 days	B	HW: 120 LW: 20	HW: 1560 mL LW: 260 mL	HW: 49 in LW: 8 in


*HW indicates the High Water treatment while LW is an abbreviation for the Low Water treatment.*

Following the isolation of strain A, a growth curve was performed to characterize the rate at which the bacterium would achieve its maximum cell density. The optical density (OD) was measured at 600 nm using a spectrophotometer every hour for 10 h. Strain A approached its maximum OD, 0.489 ± 0.005, after growing in *Pseudomonas* broth for 9 h at 30°C. Similar methods were used to characterize the growth of strain B. Strain B grew more slowly, and thus the OD was measured at 600 nm for 24 h. After 24 h strain B grew to its maximum OD of 0.389 ± 0.005. The times at which these isolates reached their maximum ODs were used in the design of the bacterial addition treatments (see section “Experimental Treatments: Water Availability and PGPR”).

### Source of Plant Material for Greenhouse Experiments

*Schizachyrium scoparium* seeds (Central Texas mix) were purchased from Native American Seed (Junction, TX, United States^2^). Seeds were stored in an air-conditioned laboratory at room temperature until they were germinated for the experimental trials.

### Experimental Treatments: Water Availability and PGPR

Drought and bacterial inoculations were conducted to understand how drought and the addition of a *Pseudomonas* isolate affect the growth of *S. scoparium*. There were two different methods in which the treatments were allocated. Method one was used for Trial 1 (14-day-old plants at the start of the experiment) and Trial 3 (70-day-old plants). Method two was used for Trial 2 (28-day-old plants). For each trial, 60 seedlings were transplanted into 1 gallon pots with a soil ratio of 3:1 potting soil and sand. The seedlings were haphazardly chosen, initial root length and shoot length were measured, and placed into the pots labeled according to treatment.

#### Treatment Method One

The bacteria treatment (B) included 10 mL of a *Pseudomonas* culture grown in *Pseudomonas* broth diluted with 10 mL of DI water (20 mL total volume). Plants that did not receive the bacterial addition treatment received sterile *Pseudomonas* broth with DI water (20 mL) that had not been cultured to act as a control. Plants that were well-watered (HW) were watered with 100 mL of water every other day. The low water treatment plants (LW) did not receive any water in addition to the 20 mL of sterile *Pseudomonas* culture or broth diluted with DI water every other day. Table 1 includes a summary of the amount of total liquid received by plants in each experimental trial.

The mean annual precipitation range of *S. scoparium* is 10–60 inches of rain per year, according to Tober and Jensen (2013). The mean annual range for the Central Texas *S. scoparium* mix used in this study is 20–40 inches (see text footnote 2). Thus, the high water treatment, which was approximately equivalent to 49 inches of rain per year (Table 1), is near the upper quadrant of the mean annual precipitation tolerance of *S. scoparium* overall, and above the range for Central Texas. By contrast, the low water treatment was roughly equivalent to plants receiving eight inches of rain per year, which is slightly below the minimum mean annual precipitation for *S. scoparium* overall, but well below the range for Central Texas *S. scoparium*.

#### Treatment Method Two

Plants receiving the bacterial addition treatment (B) received 10 mL of *Pseudomonas* culture grown in *Pseudomonas* broth every other day. The plants that did not receive the bacterial treatment received 10 mL of sterile *Pseudomonas* broth every other day. Plants that were well-watered (HW) received 15 mL of water every other day, whereas plants receiving the low water treatment (LW) received 10 mL of water every other day. The high water treatment for this trial was equivalent to approximately 10 inches of rain per year, while the low water treatment was equivalent to 8 inches of rain per year (Table 1). Thus, the high and low water treatments were just above and below the minimum mean annual precipitation for *S. scoparium* overall, and well below the minimum for Central Texas *S. scoparium*.

#### Trials

In Trial 1 *S. scoparium* seeds were germinated on May 18, 2016 on a sand bed and were misted daily. Sprouting occurred 2 weeks after the seeds were placed on a sandbed. Plants were transplanted into pots on June 11, 2016, and were misted daily until June 14, 2016 when the treatments began. Initial root length was recorded at transplanting. At the start of the experimental treatments the plants in Trial 1 were 14 days old. The treatments were applied every other day during the experiment, and continued until June 22, 2016. The total duration of treatment for this trial was 8 days (Table 1). The final shoot and root lengths, plant health, and biomass were measured on June 22, 2016. The final root lengths were recorded by measuring the length of the longest root in millimeters. Final plant health was recorded as the presence/absence of leaf rolling and discoloration, with the presence of these leaf conditions indicating reduced plant health. The types of leaf discoloration observed included yellowing and browning. In order to measure total biomass, the shoots of each plant were separated from roots by cutting the plant at the shoot base, and the roots were carefully harvested from the soil. Then, the tissues were dried in an oven at 80°C for 48 h, and weighed on an analytical balance in milligrams.

For Trial 2 *S. scoparium* seedlings were germinated on May 1, 2017 on a sand bed while misting daily. Seedlings were transplanted into pots May 22, 2017. Treatment method two and strain B were used for this trial (Table 1). The plants used for Trial 2 were 28 days old at the start of the treatments. The experimental treatments were applied to the plants starting on May 29, 2017, and occurred every other day until June 21, 2017. Dead and alive leaf count were recorded on June 7, June 14, and again at the end of the experiment on June 22, 2017. Final root and shoot length, total shoot length measurements, and final leaf counts were recorded on June 22, 2017. Similar to Trial 1, plant biomass was measured by drying and weighing the entire plant tissue (shoots and roots).

In Trial 3 *S. scoparium* seedlings were germinated on February 1, 2017 on a sand bed while misted daily. Seedlings were transplanted into pots between March 22, 2017 and April 3, 2017. Treatment method one and strain B were used for this trial (Table 1). The plants used for Trial 3 were 70 days old at the start of the treatments. The treatments began on April 12, 2017 and occurred every other day until May 6, 2017, with a treatment duration of 24 days. The number of leaves alive and dead were recorded May 3, 2017 and again at the end of the experiment on May 10, 2017. Final root length and shoot length was also measured on May 10, 2017. Similar to Trial 2, plant biomass was measured by drying and weighing the entire plant tissue.

### Statistical Analysis

In order to test the hypothesis that the effect of inoculation with a PGPR strain would interact with water availability to influence plant growth we compared final root length, final shoot length, and biomass among the four treatments. A two-way ANOVA was performed using R (R Core Team, 2017) separately for each trial to analyze the statistical significance of differences in the following parameters between the treatment groups: (1) final shoot length; (2) final root length; and (3) final total biomass. Also, for each trial, one-way ANOVA tests were paired with Tukey’s Honest Significant Difference (HSD) Tests in order to identify significant pairwise differences between the treatment groups.

To determine whether the variation in plant parameters between the three trials was due to methodological differences, a hierarchical clustering analysis was conducted on root length, shoot length, and biomass data using R (R Core Team, 2017). Then the cutree function was employed on the dendrogram generated from the hierarchical cluster analysis to determine if the plant samples were clustering based on starting age, duration of treatment, total water volume added during the experiment, and/or bacterial strain. We then performed a permutational multivariate analysis of variance (MANOVA) using the adonis function in the vegan package of R project (Oksanen, 2013) to determine if treatment had a significant effect on plant growth overall, using final root length, final shoot length, and final plant biomass as inputs to the model. In addition, another permutational MANOVA was performed using the adonis function to evaluate whether methodological differences between the trials explained a statistically significant proportion of the variability in the observed plant growth parameters across all three trials. This second MANOVA was written such that the permutations were constrained to within the treatment groups using strata as an argument in the model (Oksanen, 2013).

To determine if PGPR addition and water availability affected plant health, we tested whether leaf condition (rolling and discoloration in Trial 1, proportion of leaves alive in Trials 2 and 3) varied significantly among the treatments. For Trial 1, the format of the data was a 2 × 2 × 2 contingency table: bacteria addition (yes/no) × water treatment (high/low) × leaf condition (rolling yes/no or discoloration yes/no). We used log-linear modeling in R (loglm in the MASS package; R Core Team, 2017) to test whether there was a significant association among the number of plants with rolled or discolored leaves, bacteria treatment, and water treatment. The step function was used to select the best fitting model based on the Akaike’s Information Criterion (AIC). Rolling and discoloration were analyzed separately.

For Trials 2 and 3, we evaluated the effects of PGPR addition and water availability on plant health (proportion of senesced leaves over time) using a repeated measures ANOVA in R (R Core Team, 2017). In addition, we evaluated the individual effects and interaction between PGPR addition and water availability on plant health at individual time points using a two-way ANOVA in R (R Core Team, 2017).

## Results

### Shoot Length Differences Between Treatments and Experimental Trials

**FIGURE 1:**
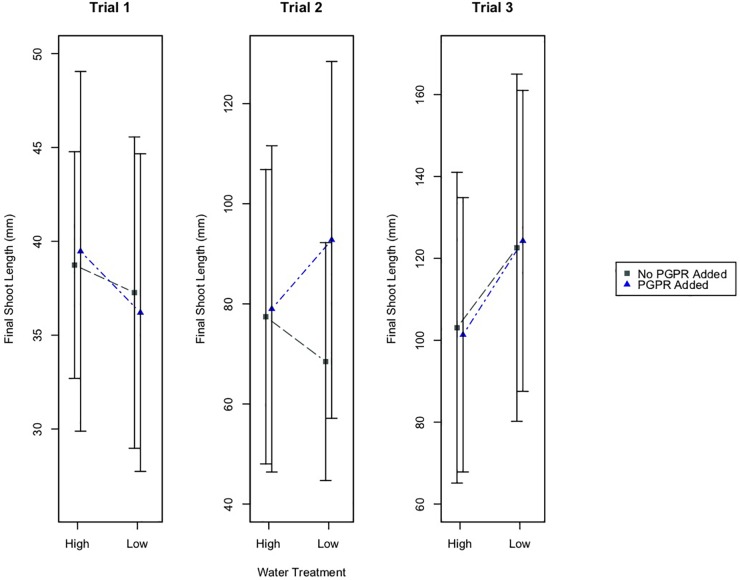
Mean final shoot length of plants, plus/minus one standard deviation, for the different water treatments (High and Low Water), and the PGPR treatments (No PGPR Added, PGPR Added). There was a statistically significant difference in the final shoot length for plants in the high water versus low water treatment in Trial 3 (*p* ≤ 0.05).

Results from two-way ANOVA tests indicate that watering treatment did not have a significant effect on shoot growth in Trials 1 and 2 (Figure 1). However, the watering treatment did have a significant effect on the final shoot lengths of plants in Trial 3 (*p* ≤ 0.05). In this trial, plants that received the low water treatment (LW) had larger shoot lengths than plants in the high water treatment (HW; Figure 1). Also, the plants that received both the low water and PGPR treatments (LWB) had larger shoot lengths than well-watered plants receiving additional bacteria (HWB; Figure 1). The PGPR treatment did not have a significant difference on final shoot length in any of the experimental trials.

### Root Length Differences Between Treatments and Experimental Trials

**FIGURE 2:**
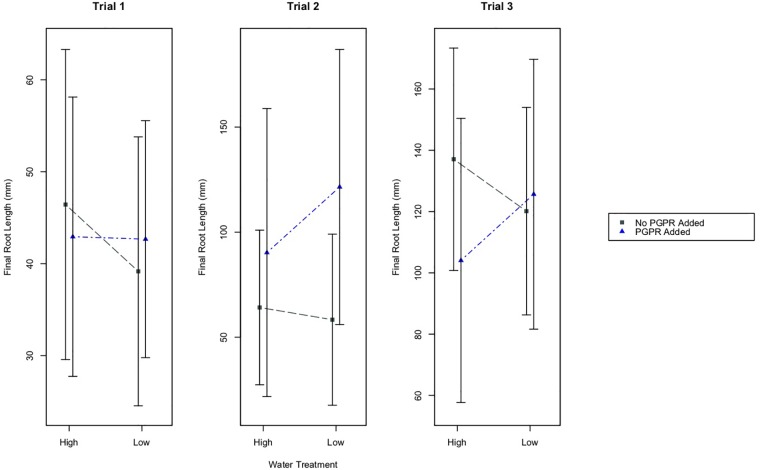
Mean final root length of plants, plus/minus one standard deviation, for the different water treatments (High and Low Water), and the PGPR treatments (No PGPR Added, PGPR Added). There was a statistically significant difference in the final root length for plants that received the PGPR treatment versus those that did not receive the addition of PGPR in Trial 2 (*p* ≤ 0.01).

The water treatments did not have a significant effect on the final root lengths of plants in any of the experimental trials (Figure 2). However, results from a two-way ANOVA identified that the PGPR treatment had a statistically significant effect on root growth in Trial 2 (Figure 2; *p* ≤ 0.01). Based on a Tukey’s HSD test, plants in Trial 2 that received the PGPR addition (LWB, HWB) had significantly greater increases in root length compared to plants that did not receive the bacterial addition (LW, HW; *p* ≤ 0.01). There were no significant effects of the PGPR treatment on the root lengths for Trials 1 and 3.

### Biomass Between Treatments and Experimental Trials

**FIGURE 3:**
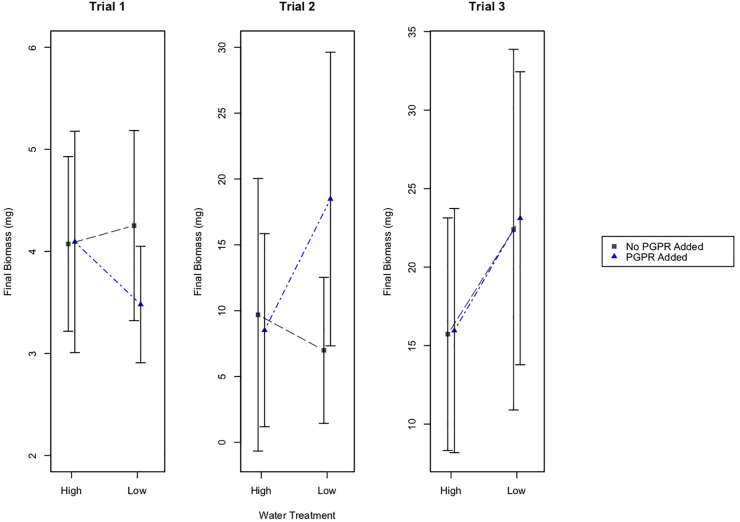
Mean final biomass of plants, plus/minus one standard deviation, for the different water treatments (High and Low Water), and the PGPR treatments (No PGPR Added, PGPR Added). In Trial 2 there was a statistically significant difference in the final biomass for plants that received the PGPR treatment versus those that did not receive the addition of PGPR (*p* = 0.05). In Trial 3 there was a statistically significant difference in the final biomass for plants in the high water versus low water treatment (*p* ≤ 0.05).

Results from a two-way ANOVA indicate that the watering treatment had a statistically significant effect on the final biomass of plants in Trial 3, where plants were 70 days old at the start of the experiment (Figure 3; *p* = 0.005). According to results from a Tukey’s HSD test, the plants in the low water treatment, with or without PGPR addition, had significantly higher mean biomass than plants in the high water treatment group (Figure 3; *p* ≤ 0.05). By contrast, the watering treatment did not have a significant effect on final plant biomass in Trials 1 or 2, where plants were much younger (14 and 28 days at the start of the experiment, respectively) at the start of the experiment.

In Trial 2, the bacterial treatment had a statistically significant effect on the final biomass of plants (two-way ANOVA; *p* = 0.05). In this trial, plants that received the PGPR addition had significantly higher biomass than plants that did not receive the additional bacteria, regardless of the watering treatment (Tukey’s HSD: *p* = 0.05). Based on the results from two-way ANOVA tests, there were no other statistically significant effects of the bacterial treatment on final plant biomass in Trials 1 or 3.

### Comprehensive Influences on Plant Growth Across Experimental Trials

The water and PGPR treatments had a statistically significant effect on plant growth overall across the trials, as identified by a MANOVA (adonis function: *F* = 7.6, *R*^2^ = 0.12, *p* ≤ 0.001). However, plants in the three trials responded very differently to the treatments. A hierarchical clustering analysis showed that the plants (based on final root length, final shoot length, and plant biomass data) form three clusters, but the plants did not cluster based on experimental treatment. Instead, the plants clustered based on experimental trial. Results from a MANOVA analysis suggest that the experimental trial (Trials 1, 2, or 3), and the experiment duration (8 or 25 days; Table 1) had statistically significant effects on overall plant growth (*F* = 119.8, *R*^2^ = 0.5, *p* ≤ 0.001). The addition of the other methodological variables, such as the bacterial strain (A or B), the total amount of water received during the experiment, and/or the amount of water received per week during the experiment, did not increase the *R*^2^ of the model output. The primary difference between each of the three experimental trials was the starting age of the plants (Table 1).

### Treatment Effects on Plant Health

**FIGURE 4:**
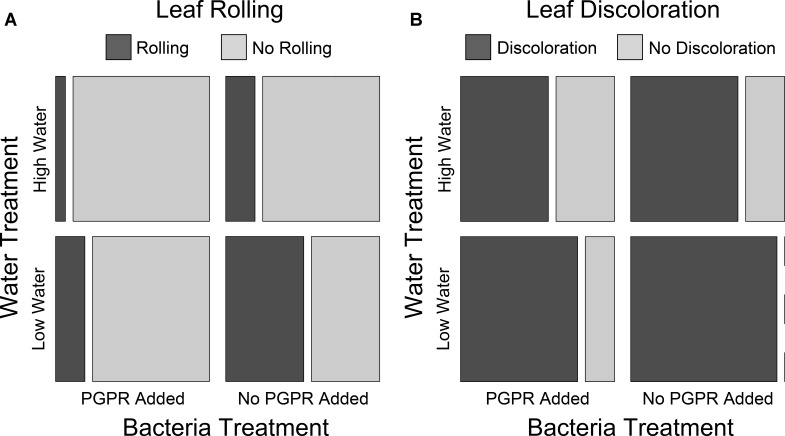
The number of Trial 1 seedlings with or without rolled **(A)** or discolored **(B)** leaves for each bacteria + water treatment combination. *N* = 15 plants for each bacteria + treatment combination; total *N* = 60 plants. Leaf condition observations were made at the end of the trial. Bacteria treatment and water treatment were both significantly associated with leaf rolling and discoloration (see main text for statistical details).

To determine whether PGPR addition and water availability impacted the health of the *S. scoparium* seedlings in Trial 1, we used log-linear modeling to test for an association between leaf condition (rolling or discoloration), bacteria treatment, and water treatment. PGPR addition and high water availability were both positively associated with reduced incidence of leaf rolling and leaf discoloration, indicating these treatments improved seedling health (Figure 4). In the mosaic plots in Figure 4, this is represented as the size of the boxes for rolled vs. not rolled (Figure 4A) or discolored vs. not discolored (Figure 4B) leaves for each combination of bacteria and water treatment. The treatment group with no PGPR addition and low water availability had the greatest proportion of plants with rolled or discolored leaves, while the group with PGPR addition and high water availability had the lowest proportions (Figure 4). Groups with either PGPR addition or high water had intermediate proportions (Figure 4). For both leaf rolling and discoloration, the model that best fit the data included two-way interactions between leaf condition and water treatment and between leaf condition and bacteria treatment, but no three-way interaction between leaf condition, bacteria treatment, and water treatment (leaf rolling best model: AIC = 12.40, LR *X*^2^ = 0.400, df = 2, *p* = 0.819; leaf discoloration best model: AIC = 14.59, LR *X*^2^ = 2.589, df = 2, *p* = 0.24). In contrast, models without interactions between leaf condition and bacteria or water treatment were poorer fits to the data (leaf rolling simple model: AIC = 17.38, LR *X*^2^ = 9.38, df = 4, *p* = 0.052; leaf discoloration simple model: AIC = 18.12, LR *X*^2^ = 10.12, df = 4, *p* = 0.038). These results indicate that water availability and *Pseudomonas* addition each had significant additive effects on leaf condition, but there were no interactive effects of water availability and PGPR on leaf condition.

**FIGURE 5:**
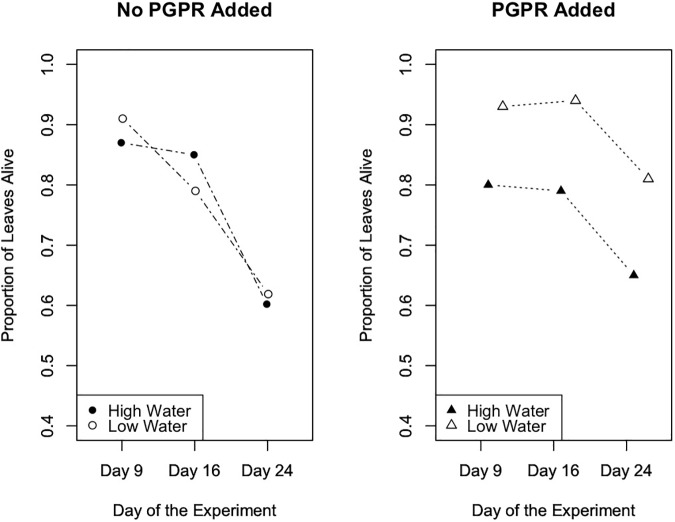
Leaf senescence, as measured by the proportion of leaves that remained alive over the course of the experiment, in Trial 2. The experimental treatments, especially the water treatments, significantly affected the number of senesced leaves (*p* = 0.003). Also, the number of senesced leaves significantly changed over time (*p* ≤ 10^-12^).

**FIGURE 6:**
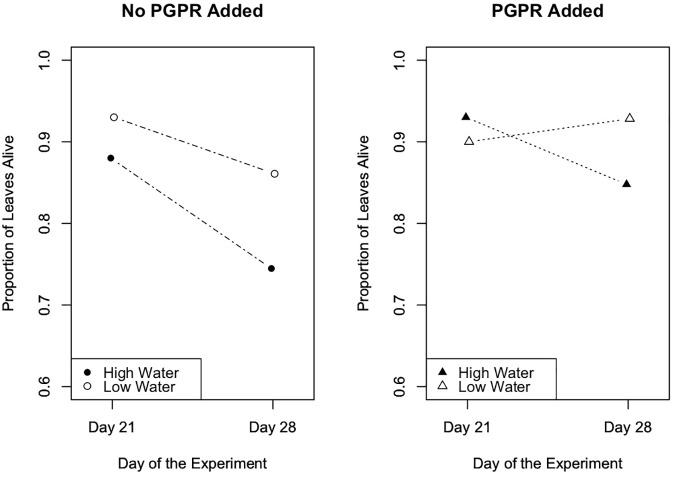
Leaf senescence, as measured by the proportion of leaves that remained alive over the course of the experiment, in Trial 3. There was a significant difference in leaf senescence between the experimental treatments (*p* = 0.02), and over time (*F* = 60.0, *p* ≤ 10^-13^). The watering treatments had a significant effect on leaf senescence throughout the experiment (*p* ≤ 0.03), while the PGPR treatment significantly affected leaf senescence at the end of the experiment (Day 28; *p* = 0.02).

To evaluate the effects of the experimental trials on the proportion of senesced leaves (plant health metric) over time in Trials 2 and 3, we performed repeated measures ANOVA tests. Results from this analysis indicated that in Trial 2 the experimental treatments significantly affected the number of senesced leaves (Figure 5; *F* = 4.7, *p* = 0.003), and the number of senesced leaves significantly changed over time (Figure 5; *F* = 89.5, *p* ≤ 10^-12^). Specifically, in Trial 2 the water treatment significantly affected the proportion of leaves that senesced over the course of the experiment (*F* = 0.2, *p* = 0.003), and there was a significant interaction between the water and PGPR treatments on leaf senescence over time (*F* = 0.1, *p* = 0.04). In Trial 3 there was also a significant difference in leaf senescence between the experimental treatments (*F* = 3.5, *p* = 0.02), and over time (*F* = 60.0, *p* ≤ 10^-13^), as well as a significant interaction between the treatments and time (*F* = 2.74, *p* = 0.05; Figure 6). Leaf senescence was significantly different between the watering treatments over the course of the experiment in Trial 3 (*F* = 6.0, *p* ≤ 0.03), whereas there was only a significant effect of the PGPR treatment on leaf senescence at the end of the experiment (Day 28; *F* = 5.4, *p* = 0.02). In both Trials 2 and 3, the greatest average amount of leaf senescence was observed for plants in the high water treatment that did not receive additional PGPR, whereas the lowest average amount of senescence was recorded for plants in the low water treatment that received the PGPR addition (Figure 5, 6).

## Discussion

Recently, there has been a considerable research focus on understanding how abiotic stress influences plant growth, as well as plant–microbial interactions, in cultivated plants, especially high-value domesticated crop species (Ström et al., 2002; Mayak et al., 2004; Zahir et al., 2008; Sandhya et al., 2010; Timmusk et al., 2011; Grönemeyer et al., 2012; Kang et al., 2014; Nadeem et al., 2014; Timmusk et al., 2014; Vurukonda et al., 2016). However, it is likely that wild plants respond differently to environmental pressures, such as water limitation, than cultivated varieties due to inherent differences in adaptive traits and selective pressures (El-Sayed et al., 2014; Milla et al., 2015; Eida et al., 2018). In addition, evidence suggests that wild plants have closer associations with their rhizobacterial partners, and that rhizobacteria have a greater effect on plant growth in native plant varieties compared to domesticated varieties (Pérez-Jaramillo et al., 2018).

The primary goal of this study was to increase our understanding of the effects of PGPR on the growth of *S. scoparium*, a wild bunchgrass species native to North America. We also evaluated whether the addition of a *Pseudomonas* PGPR influences the growth and health of *S. scoparium* in response to environmental stress, specifically differences in water availability, and whether these effects would be repeatable across different experimental trials. Since the starting age of the plants in each trial was different (14, 28, and 70 days old), we were also able to compare plant responses to PGPR and water availability across life history stages. Overall, the experimental treatments significantly affected plant growth (MANOVA: *F* = 7.6, *R*^2^ = 0.12, *p* ≤ 0.001). However, we observed significant differences between the experimental trials in terms of plant growth responses to PGPR addition and water availability, likely due to differences in the starting age of the plants and experiment duration (MANOVA: *F* = 119.8, *R*^2^ = 0.5, *p* ≤ 0.001). While the treatments had no significant effects on the growth of plants in Trial 1, there were significant effects of the treatments on plant growth in Trial 2 (starting age of 28 days) and Trial 3 (starting age of 70 days), however, plants responded differently to the treatments between these two trials. Across the trials, the water treatments and PGPR addition significantly affected plant health. Our results provide insight into how factors such as plant age and methodological choices affect the responses of a native plant species to PGPR and water availability.

### Variable Responses of *S. scoparium* to Water Availability at Different Ages

Water limitation is likely a common form of environmental stress for *S. scoparium* since it is adapted to seasonally dry habitats across North America (Tober and Jensen, 2013; PRISM Climate Group, Oregon State University, 2015; USDA-NRCS, 2019). We observed variation in how *S. scoparium* responded to differences in water availability between the experimental trials. For plants that were 14 days old at the start of the experiment (Trial 1), high water availability appeared to improve plant health, indicated by a lower incidence of leaf rolling and discoloration. Leaf rolling is a common stress response in grasses (Kadioglu et al., 2012) and *S. scoparium* is known to roll its leaves during drought (Knapp, 1984). In addition, the yellowing and browning observed was indicative of stressed and dying tissue. In contrast, for plants that were 70 days old at the start of the trial (Trial 3), the same high water treatment appeared to reduce plant health, indicated by more leaf death/senescence. High water also had a negative effect on growth in Trial 3; plants in the high water treatment had significantly shorter shoot lengths and lower biomass. These observations indicate that the low water treatment imposed drought stress on the young seedlings in Trial 1, but that high water was more stressful than low water for the older plants in Trial 3.

Differences in root system development between new seedlings and older more established plants, as well as differences between the local climate where these seeds were from and the water treatments applied, may explain these contrasting responses. According to the seed supplier, the *S. scoparium* Central Texas mix is adapted to oak woods and prairies with annual rainfalls of 20–40” (see text footnote 2). In this experiment, the low water treatment in all three trials was equivalent to 8” of rain per year, which is well below the Central Texas range. High water in Trials 1 and 3 (49” of rain per year) was above the range for the Central Texas mix. Resource availability is generally critical for plant seedling establishment (Lambers et al., 2008), and the stress response of *S. scoparium* seedlings in low water from Trial 1 is consistent with their native climate range. In addition, while *S. scoparium* seedlings are moderately tolerant to drought compared to other prairie species (Mueller and Weaver, 1942), water limitation can reduce their growth (LaGory et al., 1982). Older plants with more established root systems may be less sensitive to water limitation, but we would still expect the low water treatment in Trial 3 to be stressful. Furthermore, previous work on *S. scoparium* has observed reductions in shoot production under water limited and drought conditions (LaGory et al., 1982; Knapp, 1984; Maricle et al., 2015). Why then, would the high water treatment be more stressful for older plants? One explanation is that for older plants with more developed root systems, water levels much higher than the native precipitation range could sometimes be more detrimental than periodic water limitation. Field experiments in natural and transplanted *S. scoparium* populations indicate there may be an optimum level of soil moisture, above which there is no benefit (Knapp, 1984) and potentially even detrimental effects on growth (Rozum, 2014). Collectively, this is consistent with *S. scoparium* being tolerant of moderate, but not extreme drought, and not adapted to highly mesic environments.

Some of *S. scoparium*’s responses (or lack thereof) to water availability were more difficult to understand. First, despite effects on seedling health in Trial 1, we did not observe reduced growth of seedlings in low water. However, it is possible that the trial duration (8 days) was too short to see significant differences in biomass accumulation. Second, given that both water treatments in Trial 2 were below the precipitation range for Central Texas *S. scoparium*, it is unclear why the plants in the high water treatment (which presumably would be less stressful), had more leaf death/senescence than those in low water. In addition, there was no main effect of water availability on growth in Trial 2, despite a trial duration of 28 days. This lack of treatment effect could reflect the relatively small difference in water volume between treatments in Trial 2. Given that both treatments were well below the native precipitation range, it is likely that neither was optimal for *S. scoparium* growth.

The results from this experiment indicate that low and high water availability can impose stress on Central Texas *S. scoparium*, with effects on both plant health and growth. *S. scoparium*’s native range spans a wide precipitation gradient, including regions that experience periodic drought (Tober and Jensen, 2013). Importantly, *S. scoparium* generally does not access water in very deep soil layers (Eggemeyer et al., 2009; Mueller et al., 2013), and thus responds to water limitation through physiological adjustments to resist the negative effects of drought (Hake et al., 1984; Knapp, 1984; Maricle and Adler, 2011; Maricle et al., 2015). Its physiological response to excess water is less well studied. Collectively, this indicates that there is an opportunity for interactions between *S. scoparium* and rhizobacteria to mediate response to water stress.

### Positive Effects of PGPR on *S. scoparium* Growth and Health

Previous research has documented the plant growth promotion of rhizosphere *Pseudomonas* strains for a variety of plants growing under typical and stressed conditions (Sandhya et al., 2010; Timmusk et al., 2014; Oteino et al., 2015; Lally et al., 2017; Taketani et al., 2017; and others). In our study, surprisingly, we did not observe a consistent effect of the PGPR addition on plant growth across the three experimental trials. There was a significant positive effect of PGPR addition on root growth and biomass for plants that were 28 days old at the start of the experiment (Trial 2), but we did not observe any plant growth promotion in the other experimental trials. In fact, root growth was significantly lower for plants that received the PGPR addition for the oldest plants (70 day starting age; Trial 3). In addition, we observed that PGPR addition did not significantly affect shoot growth in any of the experimental trials under high or low water conditions. Previous research found that inoculation with PGPR strains in the *Pseudomonas* genus promoted root growth more than shoot growth (Sandhya et al., 2010). PGPR inoculum may have a greater effect on root growth than shoot growth when the interactions between PGPR strains and the plant are more localized and do not translate into systemic physiological changes within the plant. These localized interactions may be especially important under stress conditions.

Under stress, root growth is suppressed when plants produce compounds such as ethylene, and its precursor ACC, whereas many PGPR strains can reverse stress-induced growth reduction through the degradation of ACC (Yang et al., 2009). When plants experience drought, the positive effects of PGPR on root growth and biomass accumulation are especially pronounced (Jaleel et al., 2007; Timmusk et al., 2014). Root development, such as the growth of root hairs and lateral roots, may be maintained despite water limitation because PGPR create water-resistant matrices, thus allowing the root system to stay hydrated (Sandhya et al., 2009; Timmusk et al., 2014; Timmusk et al., 2015). In fact, environmental stress, such as drought, may necessitate more coordinated functions between the plants and rhizosphere microorganisms (Zang et al., 2014; Panke-Buisse et al., 2015; Naylor and Coleman-Derr, 2018). Thus, interactions between host plants and PGPR affect plants’ allocation of resources toward shoot and root growth under a range of environmental conditions.

Plants host a diversity of microbes within their native rhizosphere community and it is likely that colonization by multiple PGPR strains provides additional benefit, in terms of plant growth. Previous research has documented greater boosts in plant growth in response to inoculation with multiple PGPR strains (Mathivanan et al., 2014; Nadeem et al., 2014). In our study, we did not use sterilized soil and thus, the *Pseudomonas* isolate added during the PGPR treatments was competing for access to the plant roots. This may explain why we did not observe a more widespread effect of the PGPR addition on plant growth across our experimental trials. There is some evidence that there is a reduction in the influence of PGPR inoculation on plant growth when the native soil microbiome is intact due to competitive interactions (Çakmakçi et al., 2006; Al-Khaliel, 2010). This is possibly due to the fact that plants can be specific in the rhizobacterial partners they recruit (Vacheron et al., 2013), and other bacterial strains may be preferentially selected over the PGPR strain(s) added under experimental conditions. Also, the majority of PGPR research has been conducted with cultivated crop species, and it is possible that wild plants may interact differently with PGPR. In fact, the application of multiple PGPR strains in high concentrations had no effect on the growth of several rare wild plant species in the field, however, soil type strongly influenced plant growth (Michaelis and Diekmann, 2018). Thus, the degree to which PGPR inoculation may promote plant growth is likely dependent on plant adaptations to environmental conditions, as well as the competitive interactions between added PGPR strains and the rhizosphere microbial community.

While in our study PGPR addition seemed to have variable effects on plant growth, we did observe a measurable effect of PGPR on plant health. In Trial 1, plants that received the PGPR and high water treatments had the least amount of discoloration and rolling, while in Trials 2 and 3 leaf senescence was lowest for plants that received both the PGPR and low water treatments. By contrast, in these Trials 2 and 3 the greatest leaf senescence was measured for plants receiving only the high water treatment. The majority of similar studies focus solely on the effects of PGPR on shoot, root, or biomass yield, although a few studies have characterized PGPR effects on leaf senescence. PGPR inoculation was associated with a decline in leaf and flower senescence, as well as a delay in fruit ripening in crop species, under normal and drought conditions (Saleem et al., 2007; Ghanbari Zarmehri et al., 2013). In switchgrass and *Arabidopsis thaliana*, earlier leaf senescence was observed in plants inoculated with PGPR, however, this was attributed to an expedition of plant development in general, as opposed to a direct effect of PGPR on plant health (Poupin et al., 2013; Wang et al., 2015, 2016). Leaf senescence is often attributed to the production of ethylene and ACC in response to stress. Thus it is possible that these improvements in plant health, and declines in leaf senescence, are due to PGPR strains degrading ACC through ACC deaminase. These previous research studies have focused on cultivated and model species, and our results suggest that PGPR may confer similar plant health benefits for wild, uncultivated plant species.

### Age-Dependent Effects of PGPR on *S. scoparium* Growth

Plant age may play an important role in coordinating interactions between plants and PGPR. Results from our research suggest that younger plants may respond to water availability and the addition of PGPR differently than older plants. Low water availability may have a more detrimental effect on early seedlings (Trial 1) compared to older plants that are more established and are in a later stage of development. There is some evidence that older plants display greater resistance to stress (Hong and Hwang, 1998). The growth of the oldest plants in our study (Trial 3) also seemed to be relatively unaffected by PGPR inoculation as compared to intermediate-aged plants (Trial 2), while the growth of the youngest plants also did not appear to be affected by the addition of PGPR. In Trial 1, it is likely that the short experimental duration (Table 1) did not provide sufficient time to observe an effect of the PGPR on growth. In addition, the root microbiome membership is often more dynamic during earlier stages of development (Micallef et al., 2009; Wagner et al., 2016), which suggests the heightened importance of competitive interactions for the establishment of PGPR on the roots of young plants.

As plants age there are shifts in the secretion of root exudates (Marschner et al., 2004; Jones et al., 2009; Chaparro et al., 2014), which is an important mechanism through which plants recruit and maintain associations with rhizobacteria (Berendsen et al., 2012). Microbial species richness in the root microbiome may decrease over the plant’s lifetime (Wagner et al., 2016), and rhizosphere microbial communities often become more specialized and distinct as plants age (Roesti et al., 2006; Micallef et al., 2009). Thus, it is likely that the timing of PGPR inoculation is critical to their effect on plant growth. In our study it is possible that the oldest plants (Trial 3) had already established associations with other microbes within the rhizosphere, and the added PGPR strain was at a competitive disadvantage. The intermediate-aged plants in Trial 2 may have had a less developed microbiome at the start of the experiment, and the experiment was also long enough for a PGPR association to affect growth. In addition, the stress associated with the low water treatments could have primed the plants for PGPR association. Plants secrete different root exudates in response to environmental pressures, and these exudates select for microorganisms with functions that increase plant survival under stress (Marschner et al., 2004; Zang et al., 2014; Panke-Buisse et al., 2015; Wagner et al., 2016). The life history stage at which a plant experiences low water availability may exert strong control over the synergy between the plant and its rhizosphere microorganisms.

## Conclusion

Our study demonstrates the complex factors that influence the effects of PGPR inoculation on plant growth and plant health. Plant age, experiment duration, and water availability strongly influenced the response of *S. scoparium* to PGPR. Our results demonstrate that plant health parameters, such as leaf discoloration, rolling, and senescence, are helpful for understanding the effects of PGPR in alleviating stress. This research was distinctive in that it studied the effects of PGPR inoculation on plant growth in a wild plant species, which potentially responds differently to PGPR associations than highly domesticated crop species. Understanding the ways in which native plants respond to PGPR under a range of environmental conditions provides insight into the factors affecting the dynamics of plant–microbe interactions in an era of global change.

## Author Contributions

TB, RV, and AK conceived the idea for the study. TB, RV, and AK designed the experiment for Trial 1. RV and AK conducted the plant germination, the experimental set-up and treatment applications, and the final plant measurements for Trial 1. TB and RV conducted the bacterial isolation for all of the experimental trials, and designed and conducted the experiments for Trials 2 and 3. RV maintained and grew the bacterial cultures for all three experimental trials. TB, RV, and AK conducted data analysis and created figures for the manuscript. TB and RV created tables for the manuscript. TB and RV wrote the first three drafts of the manuscript; AK wrote sections of the manuscript. TB, RV, and AK revised, edited, and approved the final manuscript.

## Conflict of Interest Statement

The authors declare that the research was conducted in the absence of any commercial or financial relationships that could be construed as a potential conflict of interest.

## Footnotes

1 www.biolog.com

2 http://www.seedsource.com
