# Supplementary material for: Variable Influences of Water Availability and Rhizobacteria on the Growth of Schizachyrium scoparium (Little Bluestem) at Different Ages
Source: Front Microbiol. 2019 May 15;10:860. doi: 10.3389/fmicb.2019.00860 (PMC6529566; doi:10.3389/fmicb.2019.00860)
Supplement: Supplementary file 1 [file Table_1.docx]

|  | 14-day old plants | | 28-day old plants | | 70-day old plants | |
| --- | --- | --- | --- | --- | --- | --- |
|  | HW | LW | HW | LW | HW | LW |
| Root length (mm) | **30**  42.9+/-15.8 | **30**  42.7+/-15.0 | **24**  76.1+/-54.1 | **29**  88.8+/-62.0 | **29**  120.0+/-44.4 | **30**  122.9+/-38.7 |
| Shoot length (mm) | **30**  39.1+/-7.9 | **30**  36.7+/-8.3 | **26**  78.2+/-30.3 | **29**  80.2+/-32.0 | **30**  102.2+/-35.2 | **30**  123.4+/-39.0 |
| Biomass (mg) | **30**  4.1+/-1.0 | **30**  3.9+/-0.9 | **27**  11.9+/-23.9 | **29**  12.1+/-10.3 | **30**  15.8+/-7.5 | **30**  22.8+/-10.3 |

Supplemental Table 1: The number of individual plants (N; bolded), the mean plus/minus the standard deviation for all of the plants in the high water treatment (HW, with or without PGPR added) and the plants in the low water treatment (LW, with or without PGPR added).

Supplemental Table 2: The number of individual plants (N; bolded), the mean plus/minus the standard deviation for the plants that received the PGPR addition (high and low water treatments) and the plants that did not receive the PGPR addition (high and low water treatments).

|  | 14-day old plants | | 28-day old plants | | 70-day old plants | |
| --- | --- | --- | --- | --- | --- | --- |
|  | PGPR | No PGPR | PGPR | No PGPR | PGPR | No PGPR |
| Root length (mm) | **30**  39.2+/-14.1 | **30**  46.4+/-15.7 | **25**  107.9+/-67.3 | **28**  61.0+/-38.3 | **30**  114.9+/-45.8 | **29**  128.3+/-35.5 |
| Shoot length (mm) | **30**  37.8+/-9.0 | **30**  38.0+/-7.2 | **26**  86.4+/-34.3 | **29**  72.8+/-26.5 | **30**  112.8+/-36.5 | **30**  112.8+/-40.8 |
| Biomass (mg) | **30**  3.8+/-0.9 | **30**  4.2+/-0.9 | **27**  18.4+/-25.7 | **29**  8.3+/-8.2 | **30**  19.5+/-9.2 | **30**  19.1+/-10.1 |
